# Long non-coding RNA regulation of epithelial–mesenchymal transition in cancer metastasis

**DOI:** 10.1038/cddis.2016.149

**Published:** 2016-06-09

**Authors:** Q Xu, F Deng, Y Qin, Z Zhao, Z Wu, Z Xing, A Ji, Q J Wang

**Affiliations:** 1 Department of Pharmacology and Chemical Biology, University of Pittsburgh School of Medicine, Pittsburgh, PA, USA; 2 Department of Pharmacy, Zhujiang Hospital, Southern Medical University, Guangzhou, China; 3 Department of Cell Biology, School of Basic Medical Sciences, Southern Medical University, Guangzhou, China

## Abstract

Metastasis is a multistep process starting with the dissemination of tumor cells from a primary site and ending with secondary tumor development in an anatomically distant location. The epithelial–mesenchymal transition (EMT), a process that endows epithelial tumor cells with mesenchymal properties including reduced adhesion and increased motility, is considered a critical step driving the early phase of cancer metastasis. Although significant progress has been made in understanding the molecular characteristics of EMT, the intracellular mechanisms driving transition through the various stages of EMT remain unclear. In recent years, an increasing number of studies have demonstrated the involvement of long non-coding RNAs (lncRNAs) in tumor metastasis through modulating EMT. LncRNAs and their associated signaling networks have now emerged as new players in the induction and regulation of EMT during metastasis. Here we summarize the recent findings and characterizations of several known lncRNAs involved in the regulation of EMT. We will also discuss the potential use of these lncRNAs as diagnostic and prognostic biomarkers as well as therapeutic targets to slow down or prevent metastatic spread of malignant tumors.

## Facts

- EMT facilitates cancerous epithelial cells to enter into a mesenchymal-like state by endowing them migratory and invasive properties, which enables primary tumor cells to move and colonize distant organs and form secondary tumor metastases.
- EMT is closely linked to carcinogenesis, invasion, metastasis, recurrence, and resistance. Understanding the molecular mechanisms that control EMT will shed lights to the metastatic processes of tumor cells, and provide new therapeutic targets and treatment options for effective cancer therapy.
- EMT is regulated by a complex signaling network involving both transcriptional and post-transcriptional regulatory pathways related to cancer metastasis.
- LncRNAs are generally defined as non-protein-coding RNA transcripts as indicated by the lack of a discernable open reading frame. Many identified lncRNAs are polyadenylated, and locate within nuclear or cytosolic fractions.
- An increasing number of reports during the past few years support the involvement of lncRNAs in regulating tumor metastasis and progression by controlling EMT through specific ligands, receptors, or multiple EMT-related signaling pathways.
- The distribution and levels of lncRNAs in various locations, such as distal metastases, have been exploited as potential diagnostic and prognostic biomarkers for cancer.

## Open Questions

- Although there are growing interests in lncRNAs as potential biomarkers and therapeutic targets of EMT and cancer metastasis, it remains largely unknown how they are regulated in cancer cells and how they affect EMT and metastasis.
- It remains to be determined the different and precise molecular mechanisms by which functional lncRNAs switch EMT on and off during tumor development.
- Technologies should be advanced to achieve more sensitive and reliable detection and effective targeting of lncRNAs for cancer treatment.
- The effective delivery of lncRNA-targeted therapeutics to respective tumor sites, the challenges of stability, immunogenicity, and bioavailability are some of the main obstacles to be overcome in the clinical translation of lncRNA-targeted therapy.

Metastasis is responsible for as much as 90% of cancer-induced mortality, yet this process remains one of the most elusive pathological process in cancer progression.^1^ The development of new therapeutic strategies targeting key factors driving metastasis remains a challenging goal for both clinicians and scientists. First described in embryogenesis, embryonic cells undergo a process known as epithelial-to-mesenchymal transition (EMT) that allows epithelial cells to migrate and travel long distances to form tissues and organs. Once migratory embryonic cells reach their destination, they undergo the reverse process, mesenchymal-to-epithelial transition (MET) to settle, proliferate, and differentiate into different organs.^2^ Likewise, cancer cells follow the similar process to establish metastases. EMT facilitates cancerous epithelial cells to enter into a mesenchymal-like state by endowing them migratory and invasive properties, which enables a primary tumor to move and colonize distant organs and form secondary tumors metastases.^2, 3^ Moreover, these post-EMT cancer cells are often resistant to novel tumor-targeted radiotherapeutic/chemotherapeutic drugs and survive standard cancer therapies, and associate with tumor relapse and metastasis. Deeper understanding the molecular mechanisms that control EMT will not only shed lights to the metastatic processes of tumor cells, but also provide new therapeutic targets and treatment options for effective cancer therapy.

LncRNAs are commonly referred to as non-protein-coding RNA transcripts longer than 200 nt. Emerging evidence have shown that lncRNAs are dysregulated in multiple cancer types and have an important role in tumorigenesis and cancer progression.^4^ Recent studies have also demonstrated an essential role of lncRNAs in regulating EMT and cancer metastasis. In this article, we will provide a comprehensive review of the known lncRNAs relevant to EMT in cancer metastasis and discuss the molecular mechanisms underlying their regulation of EMT and their therapeutic implications as biomarkers and potential drug targets.

## Key Regulators of EMT

**Figure 1:**
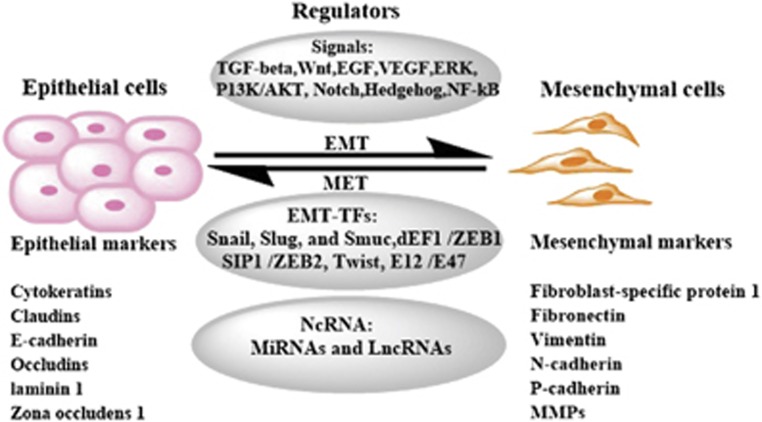
Regulatory network in EMT. EMT can be regulated by many signaling pathways, transcription factors, and transcriptional/post-transcriptional regulators.

The EMT process is accompanied by loss of epithelial marker proteins and dissolution of adherent junction proteins, such as cytokeratin, E-cadherin, *β*-catenin, *γ*-catenin, which have key roles in cell–cell adhesion.^3^ Concomitantly, mesenchymal marker proteins, such as N-cadherin, P-cadherin, fibronectin, and intermediate filament protein vimentin, are frequently overexpressed and contribute to cell migration as well as invasion-associated gene expression in different types of cancer.^5^ The most important regulators of cell adhesion are the cadherin family of proteins including E-, N-, and P-cadherin. E-cadherin as the best characterized cadherins in particular has a key role in epithelial cell–cell adhesion. It acts by binding to E-cadherins from neighboring cells and providing a physical link between their cytoskeletons.^6^ The E-cadherin is replaced by abnormal expression of N- or P-cadherin is a hallmark for EMT. The downregulation of E-cadherin leads to the release of *β*-catenin, and the latter translocates to the nucleus and functions as an activator for transcription factors, such as ZEB, Twist, Snail, and Slug, which are known to act as the repressor of E-cadherin expression. The induction of these transcription factors promotes cell migration, tissue morphogenesis, and cancer development.^7^ Other proteins that mediate EMT include vimentin and fibronectin. Vimentin and fibronectin are upregulated in cells undergoing EMT, resulting in epithelial cells to acquire a mesenchymal shape and increased motility. Fibronectin mediates cellular interactions with the extracellular matrix and is important for migration, differentiation, growth, and cell adhesion (Figure 1).^8^

## Regulations of EMT Signaling Networks in Tumor Cells

EMT is regulated by a complex signaling network at both transcriptional and post-transcriptional levels. Many growth factors, such as transforming growth factor-*β* (TGF-*β*), fibroblast growth factor (FGF), epidermal growth factor (EGF), vascular endothelial growth factor (VEGF), and their associated signaling proteins, such as nuclear factor kappa-light-chain-enhancer of activated B cells (NF-*κ*B), ERK, PI3K/AKT, Hedgehog (Hh), Notch, and Wnt, are engaged to trigger and complete an EMT process.^9^ EMT-inducing signals are cell- or tissue-type-specific and require the cooperation between multiple signaling pathways and regulators (Figure 1). These signals usually activate one of the EMT-inducing transcription factors (EMT-TFs), known as key EMT regulators, which include the Snail family of zinc-finger transcription factors (Snail, Slug, and Smuc), the dEF1 family of two-handed zinc-finger factors (dEF1/ZEB1 and SIP1/ZEB2), and the basic helix–loop–helix factors Twist and E12/E47.^3^ With the exception of Twist, these EMT-TFs repress the expression of E-cadherin by direct binding to the E-box sites in the promoter of E-cadherin, trigger gene re-programing and alter protein expression, and dynamically modulate EMT.^5^ In addition to transcription factors, EMT can be regulated by non-coding RNAs such as lncRNA and miRNA at both transcriptional and post-transcriptional levels. Previous work has revealed that EMT induction in cancer depends on an intricate network of multiple signaling pathways (Figure 1). Here we discuss several major signaling pathways that participate in the initiation of cancer EMT.

### TGF-*β* signaling pathway in EMT

TGF-*β* signaling has a predominant role in suppressing growth of normal epithelial cells, while promotes metastasis in many tumor cells, in part through tightly controlling the process of EMT.^10^ TGF-*β* activates Smads by binding to type II and type I serine-threonine kinase receptors (T*β*RII and T*β*RI), respectively. T*β*RI is phosphorylated by T*β*RII, and then activates Smads. Activated Smads translocate into the nucleus to interact with various EMT-TFs and transcriptional co-activators, and regulate the transcription of target genes. For example, TGF-*β* can activate the expression of Snail via Smads pathways as downstream effectors to repress the expression of E-cadherin and claudin.^11^ In addition to the canonical TGF-*β* /Smad pathway, TGF-*β* has been shown to regulate the expression of EMT markers through non-canonical (Smad-independent) TGF-*β* signaling pathways. It is reported that MEK/ERK, PI3K/AKT, p38-MAPK, as well as induction of miRNAs have important roles in TGF-*β*-induced EMT.^12, 13, 14, 15^

### Wnt/*β*-catenin signaling pathway in EMT

The Wnt signaling pathway is an important regulator of EMT-TFs expression and the EMT process. Wnt couples with its cell surface receptors, the low-density lipoprotein receptor and membrane protein Frizzled, to activate and stabilize the protein *β*-catenin, which is the central component of the Wnt/*β*-catenin signaling moving from the cytoplasm to the nucleus to regulate the transcription of Wnt target genes. In the nucleus, *β*-catenin acts as a coactivator of T-cell factor/lymphoid-enhancing factor-1 (TCF/LEF-1) to promote the transcription of Snail, Slug, and Twist, which in turn represses E-cadherin.^16, 17^

### Hh signaling in EMT

Studies have shown that the main role of Hh signaling pathway in tumor is to promote EMT and maintain cancer stem cells (CSCs).^18^ In humans, Hh signaling is orchestrated by two transmembrane receptors, Patched (Ptch), and Smoothened (Smo). The Hh family include three homologous Hh ligands: Sonic hedgehog (Shh), Indian hedgehog (Ihh), and Desert hedgehog (Dhh). Hh ligands can activate Ptch, release Smo, and then initiate an intracellular cascade that activates the Gli family of transcription factors. As marker of the Hh pathway activity, Gli promotes EMT by inducing the transcription of target genes such as Ptch, Wnt, and Snail.^19^ In addition, Hh signaling can cooperate with other signaling pathways such as Wnt, Notch, FGF, and TGF-*β* to modulate EMT-induced CSCs signaling network.^19, 20^

### Hypoxic/hypoxia-inducible transcription factor 1 in EMT

Recent studies indicated that each step of the metastasis process, from the initial EMT to the ultimate organotropic colonization, can potentially be regulated by hypoxia, suggesting a master regulator role of hypoxia and hypoxia-inducible transcription factor 1 (HIF-1). HIF-1 consists of an unstable *α*-subunit and a stable *β*-subunit. Under hypoxic conditions, HIF-1*α* stabilizes and translocates to the nucleus, promotes EMT by upregulating EMT-associated transcription activators or repressors, modulating EMT-associated signaling pathways, EMT-associated inflammatory cytokines, and epigenetic regulators.^21^ It has been shown that the activation of the HIF-1*α*-mediated canonical hypoxia signaling leads to the upregulation of Twist, Snail, ZEB1, and E12/E47 and enhanced EMT in breast cancer.^22, 23, 24^

## Regulation of EMT by lncRNAs

Non-coding RNAs (ncRNAs), as newcomers in genome biology, are initially regarded as transcriptional ‘noise', but accumulating evidence has demonstrated that ncRNAs such as microRNAs (miRNA), small inhibitory RNAs (siRNAs), piwi-interacting RNAs (piRNAs), circular RNAs, and lncRNAs have critical regulatory roles in gene expression.^25^ Recent studies have also elucidated the association between EMT and ncRNAs in tumor metastasis.^15, 26^

LncRNAs as a newer class of ncRNAs are divided into five broad categories: (1) sense, (2) antisense, (3) bidirectional, (4) intronic, or (5) intergenic with respect to the nearest protein-coding transcripts.^27^ Studies have demonstrated that lncRNAs are aberrantly expressed in a variety of human cancers, such as lung cancer, gastric cancer, pancreatic cancer, and breast cancer, and have important roles in various cancer-associated biological processes and signaling pathways.^28, 29^ They can act in *cis* or *trans* to modulate gene expression, for example, by binding miRNAs to protect the mRNAs.^30^ Through regulating gene expression by multiple distinct molecular mechanisms, including transcription, post-transcriptional processing, genomic imprinting, chromatin modification, and the regulation of protein function. There are growing interests in using lncRNAs for cancer diagnosis and prognosis, and lncRNAs are considered promising therapeutic targets for cancer treatment.^29^ Increasing evidence has demonstrated a potential role of lncRNAs in tumor metastasis by influence the EMT process.^29^ In the following section, we will provide an overview of the main mechanisms through which lncRNAs regulate EMT in tumor cells. A summary of EMT-associated lncRNAs and their pathophysiological functions and signaling mechanisms related to EMT is provided in Table 1.

### Role of lncRNAs in the regulation of EMT signaling networks in tumor cells

**Figure 2:**
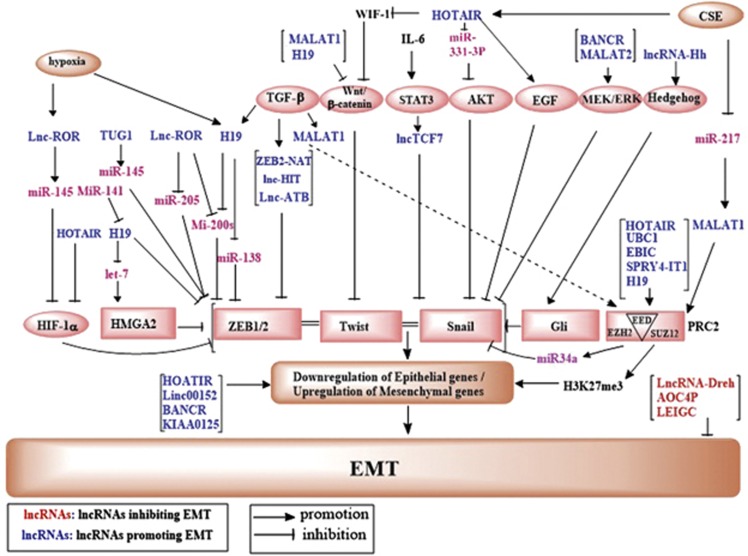
LncRNAs that are known to regulate EMT processes and their validated targets. A diagram depicts the major signaling pathways through which lncRNAs regulated EMT. Pink oval, names of the signaling pathways; pink square oval, EMT-TFs; blue text, lncRNAs that inhibit EMT; red text, lncRNAs that promote EMT; purple text, miRNAs.

LncRNAs regulate EMT through a complex network of signaling events. A diagram of the signaling network of known lncRNAs relevant to EMT is shown in Figure 2 and several main pathways are discussed below:

#### TGF-*β* pathway

TGF-*β* is a well-known EMT initiator.^31^ Lnc-ATB (lncRNA activated by TGF-*β*) is a TGF-*β*-induced lncRNA that could mediate TGF-*β*-induced EMT and has been shown to promote metastasis in hepatocellular carcinoma, colorectal cancer, gastric cancer, and breast cancer.^32, 33, 34, 35, 36, 37^ In addition, Fan and colleagues showed that TGF-*β* induced a specific lncRNA called metastasis-associated lung adenocarcinoma transcript 1 (MALAT1), which leads to EMT in bladder cancer cells. Interestingly, MALAT1 is associated with the suppression of SUZ12, which prevents the ability of Snail1 from downregulating E-cadherin.^38^ Thus, the induction of MALAT1 by TGF-*β* results in decreased E-cadherin and increased N-cadherin/fibronectin, leading to enhanced EMT.^38^ Cancer-associated fibroblasts (CAFs) as one of the principal constituents of tumor stroma have an important role in tumor development. TGF-*β*1 secreted by CAFs induces EMT of urothelial bladder cancer through lncRNA-ZEB2NAT.^39^ In addition, LncRNA-HIT (HOXA-associated transcript induced by TGF-*β*) is also involved in TGF*β*-induced EMT. The effects of lncRNA-HIT on EMT, migration, and invasion in breast cancer were rescued through introduction of ectopic E-cadherin.^40^ These findings suggest that lncRNAs can be induced by TGF-*β* and play a key role in TGF-*β*-induced EMT.

#### Hypoxia/HIF-1*α* pathway

As described earlier, HIF-1*α* regulates EMT at multiple fronts, including the expression of EMT-TFs, EMT-associated pathways, and cytokine. The oncofetal H19 lncRNA is concomitantly induced by both TGF-*β* and hypoxia in a mouse breast cancer model, which regulates E-cadherin expression and stimulates tumor metastasis through a positive feedback loop between Slug and H19/miR-675.^41^ The positive regulation of H19 by HIF-1*α* may partially explain its high expression in metastases.^42^ Linc-RoR (regulator of reprogramming) is a hypoxia-responsive lncRNA that modulates expression of miR-145 and HIF-1*α* and acts through a hypoxia/miR-145/HIF-1*α* signaling axis to modulate EMT *in vitro* and *in vivo.*^43^

#### Wnt signaling pathway

Activation of Wnt/*β*-catenin signaling pathway has been shown to induce EMT. Wnt inhibitory factor 1 (WIF-1) plays an important role in the Wnt/*β*-catenin signal pathway. Ge *et al.* demonstrated that HOX transcript antisense intergenic RNA (HOTAIR), an EMT-associated lncRNA and a powerful predictor of metastasis, inhibits WIF-1 expression and activates the Wnt pathway in esophageal squamous cell carcinoma cells.^44, 45^ HOTAIR can also directly decrease WIF-1 expression by promoting its histone H3K27 methylation in the promoter region and then activates the Wnt/*β*-catenin signaling pathway.^46^ MALAT1 can promote EMT by activating Wnt signaling *in vitro*, and knockdown of MALAT1 results in a decrease of the ZEB1, ZEB2, and Slug levels, and an increase of E-cadherin levels in bladder cancer cell or urothelial carcinoma.^47, 48^ H19 is associated with EZH2, and this association results in the activation of Wnt/*β*-catenin and subsequent inhibition of E-cadherin.^49^ Thus, Wnt signaling is an important target of lncRNAs to regulate EMT.

#### MEK/ERK pathway

The MEK/ERK pathway is another major pathway through which lncRNAs regulate EMT.^50^ Guo *et al.* demonstrated that overexpression of BRAF-activated non-coding RNA (BANCR) induces colorectal carcinoma migration by inducing EMT via the MEK/ERK signaling pathway since treatment with the MEK inhibitor affects the expression of epithelial and mesenchymal markers in colorectal cancer.^51^ Similarly, Chen *et al.* indicated that MANCR (MALAT2-activated lncRNA) contributes to gastric cancer migration by inducing EMT via a MEK/ERK-dependent mechanism as the MEK/ERK pathway inhibitor inhibits cancer metastasis.^52^

#### Hedgehog signaling pathway

CSCs are a subpopulation of neoplastic cells with self-renewal capacity and limitless proliferative potential as well as high invasive and migratory capacity. These cells are commonly associated with EMT and subsequent tumor metastasis. LncRNA-Hh contributes to Twist-induced EMT and enhances CSC-like stemness.^53^ Specifically, lncRNA-Hh transcriptionally regulated by Twist directly targets GAS1 to stimulate the activation of Hh. The activated Hh increases Gli expression, and enhances the expression of SOX2 and OCT4 to maintain CSCs. These results indicate that lncRNA-Hh impinges upon EMT and CSC stemness by modulating the hedgehog signaling.

### Coordination with PRC2

A major mechanism through which lncRNAs regulate gene expression involves the interaction with the epigenetic silencing complex polycomb repressive complex 2 (PRC2), one of the two major classes of polycomb group protein complexes. It is estimated that about 20% of all lncRNA transcripts bind PRC2.^54, 55, 56^ PRC2 that comprises EZH2, embryonic ectoderm development (EED), and suppressor of zeste 12 (SUZ12), is a histone methyl-transferase that catalyzes the trimethylation of histone H3 lysine 27 (H3K27me3) to repress transcription of specific genes such as E-cadherin.^55^ LncRNA HOTAIR has been shown to interact with PRC2 to promote cancer progression in breast cancer,^57^ gastrointestinal cancer,^58^ and hepatocellular carcinoma.^59^ Similarly, lncRNA-UBC1 (upregulated in bladder cancer 1) physically associates with PRC2 subunit EZH2 and SUZ12 and contributes to increased cancer invasion and metastasis.^60^ LncRNA H19 can also regulate bladder cancer metastasis by interacting with EZH2 and subsequently repressing E-cadherin expression and tumor metastasis.^49^ LncRNA-EBIC (EZH2-binding lncRNA in cervical cancer) promotes tumor cell invasion by binding to EZH2 and inhibiting E-cadherin expression in cervical cancer.^61^ In addition, SPRY4-IT1 (SPRY4 intronic transcript 1) is a key regulatory factor underlying the EZH2 pathway. Knockdown of SPRY4-IT1 reverses the inhibition of the EZH2 expression-mediated impairment of non-small cell lung cancer cell migration, invasion, and EMT process.^62^ Collectively, lncRNA and PRC2 interaction plays a critical role in lncRNA-regulated EMT and tumor metastasis.

### Cooperation with miRNAs

In recent years, a new regulatory mechanism has emerged that coding- and non-coding RNAs can regulate each other by competing for shared miRNA, which has been demonstrated in a variety of cancers. Abundant evidence indicates that miRNAs are capable of directly modulating EMT-TFs or EMT-activating signaling pathways.^15^ Competing endogenous RNAs (ceRNAs), also called natural microRNA sponges, are endogenous coding or non-coding transcripts including lncRNAs, circular RNAs and pseudogenes that share sequences with common microRNAs. These ceRNAs can bind and sequester miRNAs to protect their target mRNAs from being degraded.^63^ LncRNAs have been shown to regulate EMT and tumor metastasis through their ability to act as endogenous ceRNAs for EMT-regulatory miRNAs. For example, H19 promotes pancreatic cancer cell invasion and migration by increasing its target HMGA2-mediated EMT through antagonizing let-7, a microRNA and a well-known tumor suppressor in pancreatic ductal adenocarcinoma, thus H19 may repress let-7 function through competitive ceRNA network.^64, 65^ H19 also functions as a ceRNA for miR-138 and miR-200a, antagonized their functions, leading to the de-repression of their endogenous targets vimentin, ZEB1, and ZEB2 in colorectal cancer.^66^ On the other hand, miR-141 binds to H19 in a sequence-specific manner, and suppresses H19 expression and function including proliferation and invasion in gastric cancer.^67^ HOTAIR was reported to promote EMT via HER2/AKT/HSF-1/Slug pathway by inhibiting miR-331-3p in gastric cancer patients.^68, 69^ HOTAIR can also epigenetically downregulate miR34a by binding to PRC2 to activate miR34a target gene C-Met (HGF/C-Met/Snail pathway) and Snail, thereby promoting EMT in advanced stages of gastric cancer.^70^ HOTAIR also suppresses miR-568 to maintain NFAT5 expression which promotes invasion via EMT.^71^ The lncRNA TUG1 (taurine upregulated gene) can decrease the expression of miR-145 to regulate the activity of ZEB2 and EMT.^72, 73^ The family of miR-200s including miR-200a, miR-200b, miR-200c, miR-141, and miR-429 plays a key role in EMT by inhibiting EMT-TF ZEB1/2 and upregulating E-cadherin.^15, 74^ Yuan *et al.* found that lncRNA-ATB promotes metastasis of hepatoma cells through upregulating ZEB1 and ZEB2 via competitively binding to the miR-200 family members.^32^ Similarly, Lnc-ATB has also been shown to upregulate ZEB1 and ZNF-217 by competitively binding to miR-200c, leading to EMT in breast cancer cells.^36^ LncRNA UCA1 promotes bladder cancer cell migration and invasion via hsa-miR-145/ZEB1/2/FSCN1 pathway.^75^ Another lncRNA linc-ROR may function through regulating multiple miRNAs to affect EMT-associated signaling pathways. Linc-ROR modulates hypoxia signaling through a miR-145/HIF-1*α* signaling pathway in HCC cells.^43^ Similarly, linc-ROR regulates EMT by acting as a sponge for miR-205, and linc-ROR overexpression prevents the degradation of miR-205 target genes in breast cancer cells, including the EMT inducer ZEB2.^76^ In summary, miRNAs and lncRNAs can cooperate with each other in an lncRNA-miRNA functional network to regulate EMT.^77^

### Regulation of the expression of EMT-TFs and EMT markers

An increasing number of reports in the past few years support the regulation of EMT-TFs by lncRNAs, although it remains to be determined if the effects were direct. For instance, lncRNA LEIGC is a critical regulator in preventing EMT in gastric cancer, as LEIGC knockdown results in highly elevated expression of Snail, Slug, Twist and Zeb (ZEB) genes.^78^ Recent studies have also highlighted the importance of HOATIR in the regulation of EMT through regulating Snail, Slug, and Twist expression.^79^ Similarly, ZEB1-AS1 (ZEB1 antisense1) induces EMT by upregulating ZEB1 expression in hepatocellular carcinoma.^80^

EMT is marked by the loss of epithelial markers and concomitant increased expression of mesenchymal markers (Figure 1) (see Sanchez-Tillo *et al.*^9^ for a complete list of EMT markers).^8^ Many lncRNAs have been linked to the regulation of EMT through modulating EMT markers either directly or indirectly and the mechanisms remain elusive. It has been shown that HOTAIR promotes malignant transformation of lymph node stromal cells through downregulating E-cadherin and inducing EMT in normal liver stem cells.^81^ Depletion of HOTAIR increased expression of E-cadherin while concomitantly decreasing expression of vimentin and MMP9.^45^ LncRNA-Dreh regulates tumor metastasis by modifying the expression and reorganization of vimentin.^82^ Ming Sun *et al.* has also shown that lncRNA AOC4P exerts a tumor-suppressive effect on hepatocellular carcinoma tumor progression by binding to vimentin and enhancing vimentin degradation and suppressing EMT.^83^ In another study, linc00152 knockdown suppresses EMT program by decreasing N-cadherin, vimentin and oncogenic AEG-1 protein levels, and increasing E-cadherin expression.^84^ Sun *et al.* has also demonstrated that overexpression of lncRNA BANCR modulates EMT through the regulation of E-cadherin, N-cadherin, and vimentin expression.^85^

In summary, EMT is regulated by an intricate network of signaling pathways associated with cancer metastasis. Owing to the complex interactions between these signaling pathways, many of these lncRNA regulated pathways converge on a few master regulatory molecules or parallel pathways to induce changes of EMT at various levels. Thus, understanding the crosstalks between EMT-inducing signaling pathways and their regulation by lncRNAs will provide fundamental knowledge to the molecular processes of EMT.

## Therapeutic Implications

### LncRNA as potential diagnostic and prognostic biomarkers of EMT and metastasis

Metastatic spread of malignant tumors accounts for majority of cancer-related deaths. Growing efforts are devoted to the discovery of biomarkers that can be used for predicting and measuring metastatic potential of tumors. Since EMT is a potential enabling early event during metastasis, EMT regulatory lncRNAs are not only functionally important but also valuable for predicting metastasis. An increasing number of lncRNAs discovered in the past few years have been found to be dysregulated in different types of cancers and their metastases including those of breast, colon, liver, bladder, and lung, which may serve as potential biomarkers for cancer diagnosis and prognosis (see a complete list of these lncRNAs in Table 2). For example, it has been shown that the abnormal expression of HOTAIR represses several tumors and metastasis suppressor genes and has a unique association with patient prognosis.^44^ The expression levels of HOTAIR can predict tumor recurrence in HCC patients who have undergone liver transplantation therapy.^81^ MALAT1 is another prominent lncRNA overexpressed in a wide range of cancers like osteosarcoma, colorectal cancer, lung cancer, and specifically linked to high metastasis rate and poor prognosis in non-small cell lung cancer patients.^86^ Furthermore, Ying *et al.* demonstrated that MALAT1 is increased in highly invasive subline of brain metastases from lung cancer cells. The increased level of MALAT1 promotes lung cancer brain metastasis by inducing EMT, suggesting that MALATI may be a promising prognostic factor and therapeutic target for lung cancer brain metastasese.^87^ The expression of MALAT2 (metastasis-associated lung adenocarcinoma transcript 2) is upregulated in gastric cancer tissues, and a higher expression level of MALAT2 might serve as a negative prognostic marker in stage II/III gastric cancer patients.^52^ SPRY4-IT1 expression is decreased in gastric cancer tissues and associates with larger tumor size, advanced pathological stage, deeper depth of invasion, and lymphatic metastasis. Patients with lower SPRY4-IT1 expression have a relatively poor prognosis.^88^ Highly upregulated in liver cancer (HULC) serves as a specific non-invasive biomarker for HCC due to its overexpression in both tumors and plasma of HCC patients.^89^ In colorectal cancer, it is not expressed in primary tumors but is detected in colorectal cancers metastasized to liver showing its specificity for metastases.^90, 91^ HULC overexpression in gastric cancer was found to be correlated with EMT, lymph node metastasis, distant metastasis, and advanced tumor node metastasis stage and silencing of HULC effectively reversed the EMT phenotype, indicating its potential value as a prognostic factor.^92^ Similarly, high lncRNA-ATB expression is significantly associated with greater tumor size, depth of tumor invasion, lymphatic invasion, vascular invasion, and lymph node metastasis.^34^ High level of lncRNA-ATB could also predispose breast cancer patients to EMT and trastuzumab resistance.^36^ Meanwhile, lncRNA H19 expresses at high levels in human cancer tissues, but is nearly undetectable in the surrounding normal tissue, indicating the potential diagnostic value of this lncRNA. Taken together, with growing numbers of lncRNAs being discovered and characterized, their value as diagnostic and prognostic markers of EMT and metastasis will be increasingly recognized.

### Perspectives on therapeutic strategies targeting lncRNAs

With the emergence of lncRNAs as important regulators of EMT, there will be increasing demand for lncRNA-based cancer therapy. Currently, there are more than 100 clinical trials on cancer are ongoing using EMT as a keyword. These trials will help us to determine the clinical context where we could use EMT to optimize treatments for cancer patients.^93^ LncRNAs can be targeted therapeutically by a variety of approaches including (i) RNA interference (RNAi)-mediated downregulation of specific lncRNAs; (ii) antisense oligonucleotides (ASO)-based therapy; (iii) plasmid-based therapy; (iv) lncRNA mimics or small-molecule inhibitors; (v) gene therapy, and so on.^94^ Although RNAi technology offers immense therapeutic promise due to its high potency, the main obstacle of siRNA/shRNA-based therapeutics remains its poor delivery *in vivo*. In contrast to RNAi, ASOs are synthetic, short, single-stranded DNAs or RNAs (between 8 and 50 nt), another type of nucleic acid drugs, which are designed with sequence specificity to target lncRNAs. ASOs are widely used as gene knockdown reagents in tissue culture and in Xenopus, Zebrafish, and mouse model systems.^95, 96^ It has been shown that subcutaneous injection of MALAT1-targeting ASOs into a mouse xenograft model blocks the lung cancer metastasis effectively.^97^ In addition, small molecules can be synthesized to specifically bind to RNA-binding pockets of lncRNAs. They compete with protein factors or intracellular small ligands for binding of lncRNAs. Binding of small molecules may also induce conformational changes within lncRNA molecules and disrupt formation of important lncRNA structures.^98^ For instance, the interaction of HOTAIR with PRC2 or LSD1 can be inhibited with the help of HOTAIR-targeting small molecular inhibitors to reduce the metastasis in breast cancer.^99^ Overall, the development of new technologies to more efficient delivery of lncRNA-targeted therapeutics will help to bring lncRNA-based therapies closer to the clinic.

## Conclusions and Future Perspectives

EMT is a complex, multifunctional, and tightly regulated process that plays a critical role in metastatic spread of cancer cells. EMT-activating signaling pathways and downstream transcription factors are responsible for driving EMT and conferring aggressive mesenchymal phenotypes to epithelial cells. Over the past few years, lncRNAs are emerging as promising biomarkers and therapeutic targets for EMT and metastasis. Accumulating evidence has indicated that lncRNAs as a new class of ncRNAs are dysregulated to impact epithelial plasticity by targeting different signaling pathways, EMT-TFs, and EMT-related targets in a variety of cancers.^78, 100, 101, 102^ The distribution and levels of lncRNAs in various locations including distal metastases, have been exploited as potential diagnostic and prognostic biomarkers for cancer. Technologies have been advanced to achieve more sensitive and reliable detection and effective targeting of lncRNAs for cancer treatment. Despite these advances, there remain many challenges, such as limited knowledge of lncRNA functional mechanisms, targets, and binding partners, the challenges of effective delivery, stability, immunogenicity, and bioavailability of lncRNA-targeted therapeutics, which all will be tremendous tasks to undertake for the future studies. Overall, lncRNAs have shed new lights on our understanding of cancer pathways and brought our understanding of oncogenesis to a new horizon. Understanding the different and precise molecular mechanisms by which functional lncRNAs switch EMT on and off is important for opening up new avenues in lncRNA-directed diagnosis, prognosis, and therapeutic intervention against cancer.

## Figures and Tables

**Table 1:** LncRNAs that regulate EMT and their targets in different cancer types

**Cancer type**	**lncRNA**	**Function**	**Dysregulation of lncRNA**	**Potential mechanism**	**Ref.**
Bladder cancer	H19	Oncogenic	Up	Promotes EMT by interacting with EZH2 and repressing E-cadherin expression.	49
	lncRNA-ZEB2NAT	Oncogenic	Up	Induces EMT and invasion through the TGF*β*1-ZEB2NAT-ZEB2 axis in CAFs.	39
	MALAT1	Oncogenic	Up	Mediates TGF-*β* induced EMT via suz12 or promotes EMT by activating Wnt/*β*-catenin signal pathway.	38, 47
	lncRNA-HIT	Oncogenic	Up	Activated by TGF-*β* and induces EMT.	40
	KIAA0125	Oncogenic	Up	Promotes migration and invasion partly via induction of vimentin and suppression of *β*-catenin.	102
	TUG1	Oncogenic	Up	Decreases miR-145 and induces EMT.	73
	UBC1	Oncogenic	Up	Binds to PRC2 complex induces EMT.	60
	UCA1	Oncogenic	Up	Promotes migration and invasion via hsa-miR-145/ZEB1/2 /FSCN1 pathway.	75
Breast cancer	HOTAIR	Oncogenic	Up	Promotes EMT by suppressing miR-568 to maintain NFAT5 expression	71
	LncRNA-ATB	Oncogenic	Up	Activated by TGF-*β*, binds to miR-200c, upregulates ZEB1 and ZNF-217, and induces EMT.	36
	LincRNA-ROR	Oncogenic	Up	Regulates EMT by acting as a sponge for mir-205.	76
	Linc00617	Oncogenic	Up	Induces EMT via activating the transcription of Sox2.	100
	LncRNA-Hh	Oncogenic	Up	Activates the Hedgehog signaling pathway.	53
Cervical cancer	lncRNA-EBIC	Oncogenic	Up	Promotes invasion by binding to EZH2 and represses E-cadherin expression.	61
Colon cancer	BANCR	Oncogenic	Up	Induces EMT through the MEK/ERK pathway.	51
	H19	Oncogenic	Up	Promotes EMT as a ceRNA for miR-138 and miR-200a.	66
	HOTAIR	Oncogenic	Up	Not determined	45
	lncRNA-ATB	Oncogenic	Up	Not determined	34
Esophageal squamous cell carcinoma	HOTAIR	Oncogenic	Up	Inhibits WIF-1 expression and activates Wnt pathway to induce EMT.	46
Epithelial ovarian cancer	MANCR	Oncogenic	Up	Induces EMT through a MEK/ERK-dependent mechanism.	52
Gastric cancer	HOTAIR	Oncogenic	Up	Promotes EMT through regulating Snail via HER2/AKT/HSF-1/Slug pathway by inhibiting miR-331-3p or by silencing miR34a by binding to PRC2.	69, 70, 79
	H19	Oncogenic	Up	Induces EMT, promotes invasion and metastasis by binding to miR-141.	67
	HULC	Oncogenic	Up	Not determined	93
	LncRNA-ATB	Oncogenic	Up	Induces EMT, promotes invasion and metastasis through the TGF-*β*/miR-200s/ZEB axis.	33
	LEIGC	Tumor suppressor	Down	Not determined	78
	Linc00152	Oncogenic	Up	Unknown mechanism	84
	SPRY4-IT1	Oncogenic	Down	Contributes to metastasis via affecting EMT process.	88
Lung cancer	BANCR	Oncogenic	Down	Promotes EMT and metastasis by regulating of EMT marker expression.	85
	SPRY4-IT1	Oncogenic	Down	Promotes proliferation and metastasis by affecting the EMT.	62
	ZEB1-AS1	Oncogenic	Up	Induces EMT by upregulating ZEB1 expression.	80
Hepatocellular carcinoma	AOC4P	Tumor suppressor	Down	Enhances vimentin degradation and suppresses EMT.	83
	HOTAIR	Oncogenic	Up	Downregulates E-cadherin and induces EMT.	59
	H19	Oncogenic	Up	Increases HMGA2-mediated EMT through antagonizing let-7.	65
	lncRNA-ATB	Oncogenic	Up	Activated by TGF-*β*, binds miR-200s, upregulates ZEB1/2ZEB2 to induce EMT and invasion.	32
	LncRNA-Dreh	Tumor suppressor	Down	Inhibits metastasis by repressing vimentin expression and changing the normal cytoskeleton structure.	82
	linc-RoR	Oncogenic	Up	Involves in miR-145/HIF-1α signaling module.	43
	lncTCF7	Oncogenic	Up	Acts through IL-6/STAT3/lncTCF7 signaling axis leading to HCC aggressiveness through EMT induction.	101

**Table 2:** Overview of clinical lncRNA biomarkers relative to EMT in cancer metastasis

**Cancer type**	**lncRNA**	**Biomarker usability potential**	**Ref.**
Bladder cancer	UBC1	High expression of UBC1 confers a worse prognosis, lymph node metastasis, and survival.	60
Breast cancer	lncRNA-ATB	High expression of LncRNA-ATB in breast cancer patients confers EMT and trastuzumab resistance.	36
Cervical cancer	lncRNA-EBIC	High expression of lncRNA-EBIC is associated with a recurrence and worse prognosis.	61
Colon cancer	BANCR	Overexpression of BANCR is associated with high lymph node metastasis and high tumor stage.	51
	lncRNA-ATB	Overexpression of lncRNA-ATB confers bigger tumor size, and associates with high lymph node and hematogenous metastasis.	34, 37
	HOTAIR	High expression of HOTAIR is associated with high metastasis and worse prognosis.	45
Epithelial ovarian cancer	HOTAIR	High expression of HOTAIR is associated with a worse prognosis.	44
Esophageal cancer	HOTAIR	High expression of HOTAIR is associated with a worse prognosis.	46
Gastric cancer	MANCR	High expression of MALAT2 is associated with a worse prognosis in stage II/III.	52
	lncRNA-ATB	High expression of LncRNA-ATB is associated with a worse prognosis.	33
	HULC	Overexpression of HULC is associated with high lymph node metastasis.	92
	Linc00152	Overexpression of Linc00152 is a diagnostic indicator of gastric cancer.	84
	SPRY4-IT1	Low expression of SPRY4-IT1 confers a worse prognosis.	88
	HOTAIR	High expression of HOTAIR is a predictor of recurrence liver transplantation.	79
Hepatocellular Carcinoma	HULC	Overexpression of HULC is associated with high lymph node metastasis.	89, 90
	linc-RoR	Overexpression of linc-RoR is a diagnostic indicator of HCC and chemoresistance.	43
	ZEB1-AS1	High expression of ZEB1-AS1 confers a worse prognosis.	80
Lung cancer	BANCR	Low expression of BANCR confers a worse prognosis.	85
	MALAT1	Overexpression of MALAT1 is associated with high lung cancer brain metastasis.	86, 87
	SPRY4-IT1	Low expression of SPRY4-IT1 confers a worse prognosis.	62

## Abbreviations

ASOs: antisense oligonucleotides
BANCR: BRAF-activated non-coding RNA
CAF: cancer-associated fibroblasts
ceRNAs: competing endogenous RNAs
CSCs: cancer stem cells
EGF: epidermal growth factor
EMT: epithelial-mesenchymal transition
EMT-TFs: EMT-inducing transcription factors
EZH2: enhancer of zeste homolog 2
FGF: fibroblast growth factor
HGF: hepatocyte growth factor
HGF/SF: hepatocyte growth factor/scatter factor
Hh: hedgehog signaling
HIF-1*α*: hypoxia-inducible factor-1*α*
HULC: highly upregulated in liver cancer
H3K27me3: histone H3 lysine 27 trimethylation
lncRNAs: long non-coding RNAs
lncRNA TUG1: taurine upregulated gene
lncRNA-UBC1: upregulated in bladder cancer 1
MALAT1: metastasis-associated lung adenocarcinoma transcript 1
MALAT2: metastasis-associated lung adenocarcinoma transcript 2
MALAT2 MANCR: MALAT2-activated lncRNA
MET: mesenchymal-to-epithelial transition
miRNA: microRNA
ncRNAs: non-coding RNAs
NF-*κ*B: nuclear factor kappa-light-chain-enhancer of activated B cells
PRC2: polycomb-repressive complex
RNAi: RNA interference
SPRY4-IT1: SPRY4 intronic transcript 1
TF: transcription factors
TGF-*β*: transforming growth factor-*β*
T*β*RII and T*β*RI: type II and type I serine–threonine kinase receptors
UBC: urothelial bladder cancer
VEGF: vascular endothelial growth factor
WIF-1: Wnt inhibitory factor 1
ZEB1-AS1: ZEB1 antisense 1
